# Feasibility and Robustness of 3T Magnetic Resonance Angiography Using Modified Dixon Fat Suppression in Patients With Known or Suspected Peripheral Artery Disease

**DOI:** 10.3389/fcvm.2020.549392

**Published:** 2020-10-30

**Authors:** Karl Jakob Weiss, Holger Eggers, Christian Stehning, Marc Kouwenhoven, Mithal Nassar, Burkert Pieske, Philipp Stawowy, Bernhard Schnackenburg, Sebastian Kelle

**Affiliations:** ^1^Department of Internal Medicine/Cardiology, German Heart Institute Berlin, Berlin, Germany; ^2^DZHK (German Centre for Cardiovascular Research), Partner Site Berlin, Berlin, Germany; ^3^Philips Research, Hamburg, Germany; ^4^Philips Healthcare, Hamburg, Germany; ^5^Philips Healthcare, Best, Netherlands; ^6^Sackler School of Medicine, Tel Aviv University, Tel Aviv, Israel; ^7^Department of Cardiology, Rabin Medical Center - Beilinson Hospital, Petach Tikva, Israel; ^8^Department of Internal Medicine/Cardiology, Charité Virchow Klinikum, Berlin, Germany

**Keywords:** magnetic resonance angiography, peripheral artery disease, 3 Tesla, modified Dixon (mDixon), image quality, signal-to-noise ratio, vessel-to-background contrast, fat suppression MRI

## Abstract

**Objective:** Contrast-enhanced magnetic resonance angiography (CE-MRA) is a well-established non-invasive imaging technique for the assessment of peripheral artery disease (PAD). A subtractionless method using modified Dixon (mDixon) fat suppression showed superior image quality at 1.5T over the common subtraction method, using a three-positions stepping table approach with a single dose of contrast agent. The aim of this study was to investigate the feasibility of subtractionless first-pass peripheral MRA at 3T in patients with known or suspected PAD and to compare the performance in terms of vessel-to-background contrast (VBC), signal-to-noise ratio (SNR), and subjective image quality to conventional subtraction MRA.

**Methods:** Ten patients [mean age 69 years ± 12 standard deviation (SD)] with known or suspected PAD were examined on a clinical 3T scanner (Ingenia, Philips Healthcare, Best, Netherlands) at three table positions using subtractionless and subtraction first-pass peripheral MRA. Two readers rated image quality on a four- point scale. Interobserver agreement was expressed in quadratic weighted κ values. VBC was assessed with a semi-automated process and SNR was compared in a healthy volunteer.

**Results:** Subjective image quality was significantly better with the subtractionless method overall (mean image quality for mDixon imaging: 2.88 ± 0.32 SD vs. for subtraction imaging: 2.57 ± 0.48 SD; *P* < 0.001) and per table position (abdominal position: 2.88 ± 0.32 vs. 2.57 ± 0.48 SD; *P* < 0.001); upper leg position: (2.97 ± 0.15 SD vs. 2.68 ± 0.37 SD; *P* < 0.001; lower leg position: 2.60 ± 0.50 SD vs. 2.13 ± 0.60 SD; *P* < 0.001). Vessel-to-background contrast increased by 22% with the subtractionless method overall (mean VBC for mDixon imaging: 23.16 ± 8.4 SD vs. for subtraction imaging: 19.00 ± 8.1 SD; factor 1.22, *P* < 0.001). SNR was 82% higher with the subtractionless method (overall SNR gain 1.82; *P* < 0.001).

**Conclusion:** This study demonstrated the feasibility and robustness of subtractionless first-pass peripheral MRA at 3T in patients with known or suspected PAD using a three- positions stepping table approach with a single dose of contrast agent. It showed increased image quality compared to the conventional subtraction method and superior performance in terms of SNR and vessel-to-background contrast.

## Introduction

Contrast- enhanced magnetic resonance angiography (CE-MRA) is a well-established, highly trusted imaging technique for the non-invasive assessment of peripheral artery disease (PAD) (1–3). It involves corresponding 3D acquisitions before and during the initial arterial passage of a contrast agent to suppress background signal by subtraction, typically at three or four table positions to cover the vascular tree from the infrarenal aorta down to the feet (4, 5).

This stepping table subtraction method is available for clinical use from all major vendors of MR systems but suffers from several intrinsic drawbacks. First, a subtraction for background suppression is prone to misregistration artifacts resulting from patient movement, including involuntary (such as peristaltic) motion (6). Furthermore, a subtraction is intrinsically associated with a decrease in signal-to-noise ratio (SNR) by a factor of √2 because of unfavorable noise propagation (7).

Recently, a clinical study employing a subtractionless method was performed at 1.5T in patients with suspected PAD (8), which involves a modified Dixon (mDixon) sequence with a multigradient echo acquisition and a relatively flexible choice of echo times (TEs) (9). This method uses reconstructed water-only images to reduce background signal which originates predominantly from lipid signals. Advantages of the subtractionless method over the subtraction method were shown in terms of SNR and vessel-to-background contrast (VBC), robustness to motion, and scan time, in both theory and practice (8).

Due to the large chemical shift between water and lipids at 3T, a better selection of echo times that allows for both short scan times and efficient water- fat separation is possible. In theory, this may yield a further improvement in SNR, as the reconstruction of water-only images uses data from two independent echoes in an approximately optimal fashion.

The depiction of the vessel lumen against the background is key in all CE-MRA variants. However, a manual evaluation of VBC based on user-defined regions-of-interest (ROIs) in angiograms is very cumbersome and, to some extent, user-dependent. In order to simplify the workflow and reduce user dependence, a tool for semi-automated VBC analysis was developed and employed.

The purpose of this work was hence to investigate the feasibility of subtractionless first-pass peripheral MRA at 3T in patients with known or suspected PAD using a three- positions stepping table approach with a single dose of contrast agent and to evaluate the performance in terms of SNR and VBC, as well as subjective image quality, compared to the conventional subtraction method.

## Methods and Materials

### Theory

The resulting SNR in a subtraction image is given by

$$\begin{array}{l} SNR_{S}=\frac{S_{2}-S_{1}}{\sqrt{2}\;\sigma } \end{array}$$

where S1 and S2 are the signal and σ is the standard deviation of the noise in the pre- and post-contrast images, respectively. Since the noise in the pre- and post-contrast images is uncorrelated, the standard deviation of the noise simply scales with the square root of two in the subtraction image. However, unlike in averaging, the signal does not double. At best, the signal in the vasculature in the pre-contrast image is negligible due to the long T1 of unenhanced blood and the short TR and high flip angle employed, leading to strong signal saturation. In an exemplary measurement in the abdominal aorta the pre-contrast signal intensity was below 10% of the post-contrast signal intensity and was therefore considered negligible. Consequently, the subtraction decreases the SNR by at least the square root of two, or more if the pre-contrast signal intensity is significant, or if a complex subtraction is employed and the signal phases of the pre- and post-contrast images interact in an unfavorable fashion.

In contrast, the gain in SNR in a water-only image reconstructed with a subtractionless method involving two post-contrast acquisitions at different echo times TE_1_ and TE_2_ is given by

$$\begin{array}{l} SNR_{D}=\frac{S}{\sigma }\sqrt{1-cos\left(2\pi \;\Delta TE\;\Delta f_{F}\right)} \end{array}$$

where ΔTE = TE_2_-TE_1_ denotes the echo spacing and Δf_F_ denotes the offset of the resonance frequency of fat relative to water, which is ~ −421.5 Hz at 3T, and *S* denotes a representative signal in the post-contrast images (9). Depending on the chosen ΔTE, the SNR may increase at most by the square root of two. In terms of SNR, this renders the behavior of the water-fat separation comparable to that of averaging. Noteworthy, the noise propagation in the water-fat separation is, in the present application, much more favorable at 3T than at 1.5T, as typical echo times spacings, which are mostly defined by the desired resolution and available gradient performance, are close to the optimum of 1/(2^*^Δf_F_), yielding an SNR increase approximately equal to the square root of two.

Overall, the achievable gain in SNR using a subtractionless mDixon reconstruction instead of a conventional subtraction reconstruction amounts to a factor of 2. This factor is composed of a √2 gain by omitting the subtraction and a √2 gain by the water-fat separation and an appropriate choice of echo times. The theoretical SNR gains obtained for each table position with the employed sequence parameters are summarized in Table 1.

**Table 1:** Echo times per anatomical location and respective SNR gains by the water-fat separation (SNR) and by eliminating the subtraction (Total SNR).

**Anatomical location**	**TE1/TE2 (ms)**	**SNR**	**Total SNR**
Abdominal position	1.48/2.84	1.38	1.95
Upper leg position	1.51/2.83	1.39	1.97
Lower leg position	1.58/2.88	1.40	1.98

*TE1/TE2, Echo times in millisecond; SNR, Signal-to-noise ratio by separation; Total SNR, total gain in signal-to-noise ratio compared with subtraction method*.

### MR Sequence and Patient Cohort

Ten patients with known or suspected PAD and clinically indicated MRA were examined on a clinical 3T scanner (Ingenia, Philips Healthcare, Best, Netherlands). Exclusion criteria included common contraindications for magnetic resonance angiography as well as refusal or inability of the patients to get through the examination. During and after injection of 10 ml Gadovist (Bayer Healthcare, Berlin, Germany) at 0.5 ml/s, contrast-enhanced images were acquired successively at three table positions, each with a field of view (FOV) of 430 × 400–450 × 180–200 mm^3^, using a 3D T1-weighted spoiled dual-gradient-echo sequence with a TE1/TE2/TR of 1.5–1.6/2.8–2.9/4.4–4.7 ms. The measured spatial resolution increased from 1.3 × 1.3 × 17 mm^3^ at the first, abdominal position to 1.0 × 1.0 × 1.5 mm^3^ at the third, lower leg position. Scan times ranged from 17 os for the first position to 23 s for the third position, with an up to 8-fold acceleration by SENSE and a partial Fourier factor of 0.7. RF shimming was performed individually at each position. A direct comparison between the subtraction and subtractionless methods was enabled by additionally collecting corresponding non-contrast-enhanced images before injection using the same sequence.

Water images were reconstructed from the contrast-enhanced images using mDixon with a multi-peak spectral model of fat (9) and subtraction images were generated from the first gradient-echo (TE_1_) images before and after contrast administration. For visualization, coronal MIPs were calculated for each position and were stitched together to reach a virtual FOV of 1,210 mm in FH direction. One healthy volunteer was additionally examined without administration of contrast agent or radiofrequency pulses for SNR analysis.

All image acquisition was part of routine clinical practice. Conduct and reporting of this study were carried out in accordance with the Helsinki Declaration as revised in 2013. The studies involving human participants were reviewed and approved by the ethics committee of the Charité—Universitätsmedizin Berlin. Both the institutional data protection office and the local ethics committee waived the need for informed consent and provided permission to analyse the anonymized images obtained with the imaging protocol as described below.

### Image Quality Analysis

We prespecified 23 clinically relevant vessel segments that were assessed with regard to image quality by two cardiologists (3 and 6 years of experience, respectively), each blinded to the rating of the other. Segments were rated based on a four-point scale: 0 = not evaluable, no arteries visible (non-diagnostic); 1 = poor to moderate quality, not all arterial segments evaluable due to noise, heterogeneous vascular enhancement or poor fat suppression (partly non-diagnostic); 2 = acceptable quality but some noise or heterogeneous signal, all arterial segments evaluable for diagnostic purposes; 3 = good quality, all arterial segments evaluable for diagnostic purposes without artifacts.

### Vessel-to-Background Contrast Analysis

The volumetric data was reformatted into the axial view, and cylindrical regions of interest (ROIs) centered on the main vessels were manually defined in the mDixon images and labeled according to the respective vessel segment. For each of the 23 vessel segments, 3 different ROIs were evaluated resulting in a total of 69 ROIs per patient and a total of 690 ROIs. The ROIs were copied across to the corresponding subtraction images without modification.

In order to simplify the workflow and reduce user dependence, an automated algorithm to derive the VBC from each ROI was employed, where the VBC was defined as:

$$\begin{array}{l} VBC=\frac{mean_{vessel}-mean_{background}}{\sigma_{background}} \end{array}$$

The algorithm segmented each ROI in the mDixon images into vessel and background by first searching for the 10 most intense local maxima, where the maxima were sorted by distance to the ROI isocenter and the innermost maximum was identified as a seed point for the vessel. The vessel lumen was then determined and grouped using a flood fill algorithm. From the remaining voxels, vessel branches, and other hyperintense areas were removed in order to approximate the background. The resulting segmentation was identically applied to the mDixon and the subtraction images.

### Signal-to-Noise Ratio Analysis

**Figure 1:**
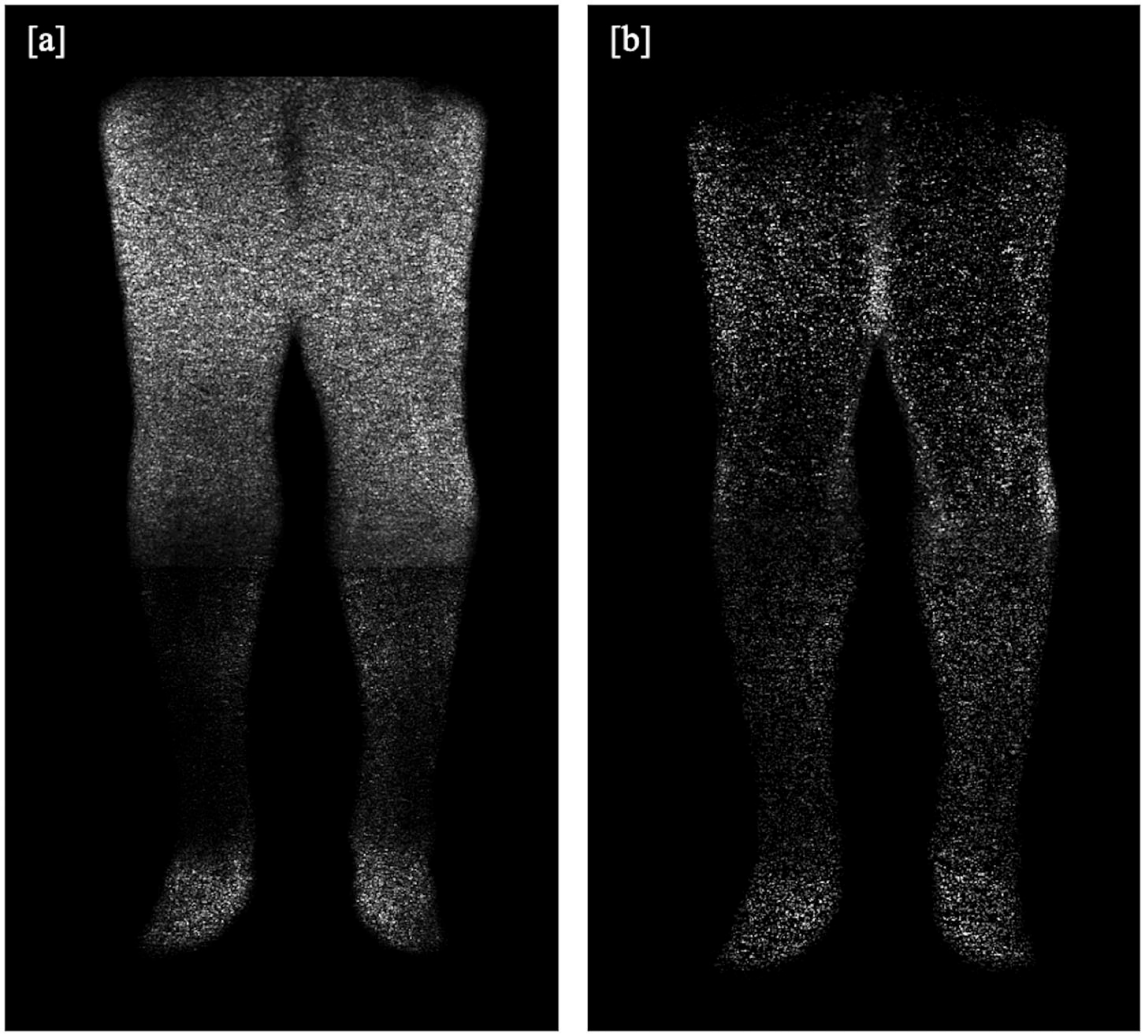
**(a,b)**: Additional scan in a healthy volunteer for SNR analysis, without application of a contrast agent or radiofrequency excitation, providing **(a)** a subtraction image and **(b)** an mDixon water or fat-suppressed image.

As described in previous studies (8), a numerical analysis of the SNR based on the actual angiograms is difficult, user-dependent, and strongly depends on the measurement location, especially in images produced with a parallel imaging reconstruction (10). We assumed that the intravascular signal intensity of post-contrast images does not differ between the mDixon and the subtraction method (8) and that the signal of pre-contrast images is negligible. Therefore, the gain in SNR was estimated based on an additional scan in a healthy adult volunteer, in which typical CE-MRA sequence parameters were employed, except that the radiofrequency excitation was disabled, and no contrast agent was administered in order to obtain noise-only images (11). As shown in Figures 1a,b, the noise level is highly dependent on location and other sequence parameters (e.g., spatial resolution and acceleration factor of the respective imaging position). We measured the SD of noise using the exact same ROIs as in one of the patients to evaluate the relative SNR gain at typical vessel segment locations. We then calculated mean SD for each method and the relative SNR gain which corresponds to the ratio of standard deviations in the noise-only images was determined over all locations.

### Statistical Analysis

Values were tested for normal distribution using Shapiro–Wilk test. Normally distributed values such as VBC, SNR, and image quality scores are reported as means ± standard deviation and Student's *t*-test was used to compare means. Non-normally distributed variables are reported as median ± interquartile range and compared using Mann–Whitney *U*-test. Agreement regarding image quality analysis was expressed in quadratic weighted kappa values (κ) (12). A κ-value of 0 indicated poor agreement; 0.01–0.20 slight agreement; 0.21–0.40 fair agreement; 0.41–0.60 moderate agreement; 0.61–0.80 good agreement; 0.81–1.0 excellent agreement. Significance was assumed at *P*-values smaller than 0.05. All statistical analyses were performed using SPSS 27.0 (IBM, New York, NY, USA).

## Results

### Image Quality and Feasibility

All 10 patients (4 female, 6 male) were successfully scanned. Median patient age was 69 years (interquartile range 68–74 years), mean body mass index was 29.0 ± 3.6 kg/m^2^, resulting in a mean contrast agent dose of 0.123 ± 0.013 mmol/kg per patient. The overall mean image quality score ± standard deviation was 2.88 ± 0.32 for the mDixon method and 2.57 ± 0.48 for the subtraction method (*P* < 0.001). The mean image quality score was significantly worse in the lower leg position compared with the abdominal and upper leg position for both methods (*P* < 0.001). Throughout all table positions, the mean image quality score was significantly better with the mDixon method than with the subtraction method (Table 2).

**Table 2:** Subjective image quality for the mDixon and subtraction methods averaged for both readers.

**Anatomical location**	**Imaging method**	**Mean score ± SD**	***P-*value**
Total	mDixon	2.88 ± 0.32	
	Subtraction	2.57 ± 0.48	<0.001
Abdominal position	mDixon	2.99 ± 0.08	
	Subtraction	2.75 ± 0.28	<0.001
Upper leg position	mDixon	2.97 ± 0.15	
	Subtraction	2.68 ± 0.37	<0.001
Lower leg position	mDixon	2.60 ± 0.50	
	Subtraction	2.13 ± 0.60	<0.001

*SD, Standard deviation; Score: 0, not evaluable, no arteries visible (non-diagnostic); 1, poor to moderate quality, not all arterial segments evaluable due to noise, heterogeneous vascular enhancement or poor fat suppression (partly non-diagnostic); 2, acceptable quality but some noise or heterogeneous signal, all arterial segments evaluable for diagnostic purposes; 3, good quality, all arterial segments evaluable for diagnostic purposes without artifacts*.

For the subtraction method, one reader rated 7 of 230 vessel segments (3.04%) to be partly (five segments) or completely (two segments) non-diagnostic, the other reader rated 10 vessel segments to be partly non-diagnostic (4.35%). Noticeably, of the 14 segments that were rated with a score of 1 or 0 by at least one reader, only 3 segments were deemed partly or completely non-diagnostic by both readers.

**Figure 2:**
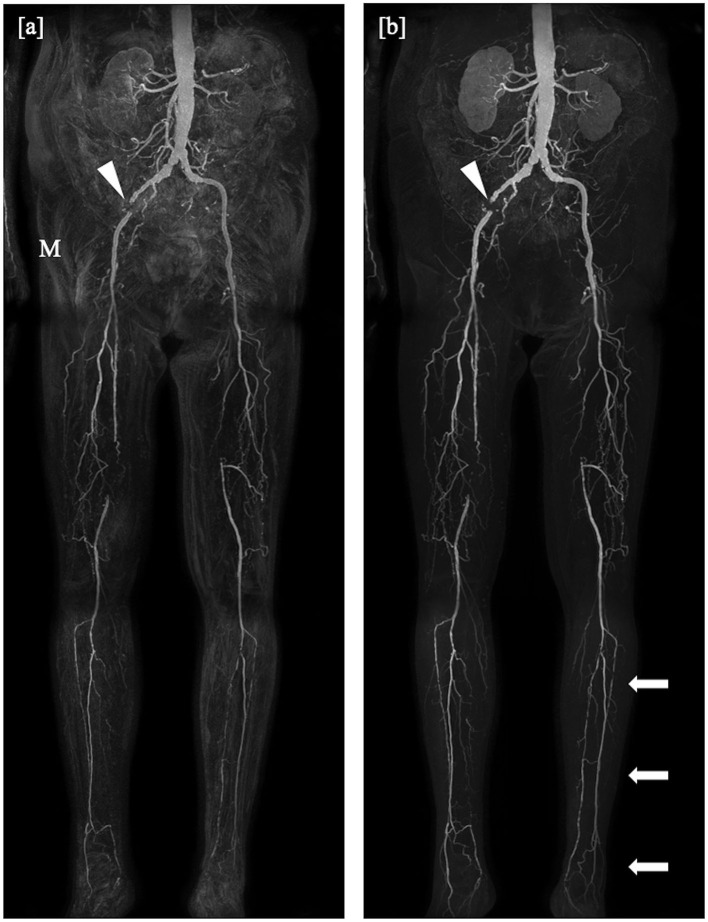
Coronal maximum intensity projections obtained by the subtraction method **(a)** and the subtractionless mDixon method **(b)** show bilateral occlusion of the superficial femoral artery in the distal third in the right leg and directly at the offspring in the left leg, on both sides bridged by collaterals, and a total occlusion of the left iliac internal artery. Note the misregistration artifacts (M) in the subtraction image as compared to the mDixon image, possibly due to movement of the patient or bowel motility and the better depiction of the distal arteries of the lower leg in the mDixon image (arrows). Stent artifacts occur regardless of imaging method (arrowheads).

**Figure 3:**
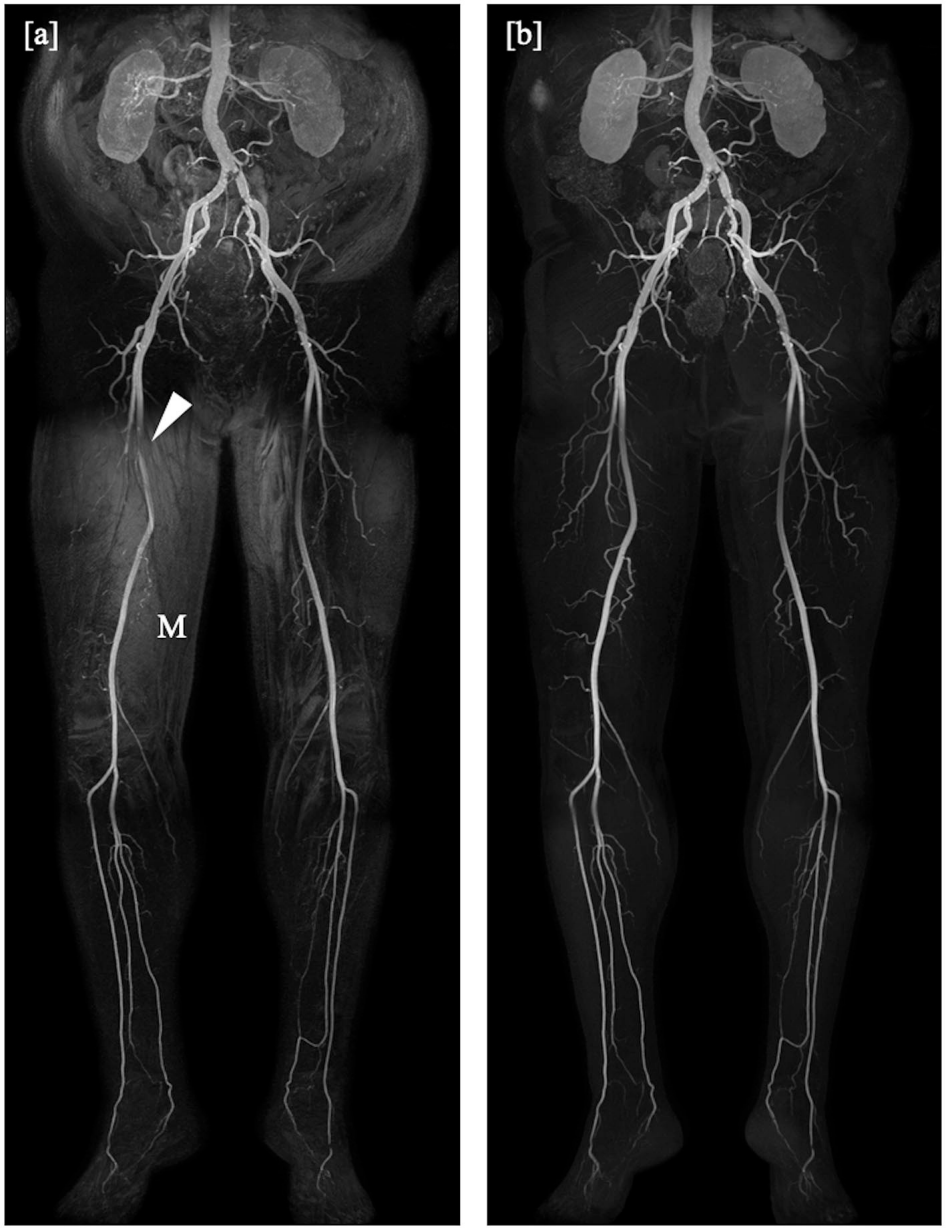
Coronal maximum intensity projections obtained by the subtraction method **(a)** and the subtractionless mDixon method **(b)** show bilateral stenoses of the common iliac artery and an occlusion of the left internal iliac artery, as well as an extensive occlusion of the left posterior tibial artery. Note the misregistration artifacts (M) in the subtraction image as compared to the mDixon image. The right superficial femoral artery was rated occluded in the subtraction image whereas it is clearly patent on the mDixon image (arrowhead). Note also the better visibility of the profound femoral arteries on both sides.

For the mDixon method, one reader considered all vessel segments evaluable for diagnostic purpose, whereas the other reader rated three vessel segments to be non-diagnostic (1.3%). Non-diagnostic vessel segments were predominantly found in the lower leg table position, mostly due to misregistration artifacts (see Figures 2, 3) and excessive background noise in the subtracted images. A total of nine vessels were rated as stenotic or occluded in subtraction images, but as patent in corresponding mDixon images, eight of which were in the lower leg position and one in the upper leg position. An example is shown in Figure 3.

Overall, interobserver agreement with regard to subjective image quality showed moderate agreement with a κ value of 0.534 and an acceptable reliability with a Cronbach's alpha of 0.704 (13). On a per table position analysis, agreement for the mDixon method was good for both the upper leg position and the lower leg position with kappa values of 0.66 and 0.72, respectively. Mean image quality and interrater agreement for all table positions is presented in detail in Table 3a. Both raters showed very high agreement for the abdominal position images obtained by the mDixon method; however, the resulting kappa value was very low, possibly due to high congruence of the two ratings (14). Actual ratings are presented in Table 3b. For the subtraction method, interobserver agreement was considerably lower, with an actual disagreement embodied by a negative kappa value for the abdominal position (Table 3c).

**Table 3a:** Subjective image quality per anatomical location and observer.

	**Anatomical location**								
	**Abdominal position**			**Upper leg position**			**Lower leg position**		
	**Rater 1**	**Rater 2**		**Rater 1**	**Rater 2**		**Rater 1**	**Rater 2**	
mDixon (mean ± SD)	2.98 ± 0.13	2.99 ± 0.09		2.96 ± 0.19	2.98 ± 0.14		2.53 ± 0.60	2.67 ± 0.48	
*K-*value			–^*^			0.66			0.72
Subtraction (mean ± SD)	2.88 ± 0.32	2.63 ± 0.49		2.81 ± 0.40	2.56 ± 0.50		2.20 ± 0.71	2.05 ± 0.65	
*K-*value			0.09			0.30			0.55
*P*-value	0.002	<0.001		0.014	<0.001		0.006	<0.001	

Quadratic weighted K-values represent inter- rater reliability, p-values are given for differences in image quality between the mDixon and subtraction methods. SD, Standard deviation; K-value: weighted (quadratic) Cohen's kappa;

* *Calculation of a plausible kappa value is not possible due to extreme margins caused by extreme agreement. The calculated kappa values for the abdominal position are negative and shown in Tables 3b,c together with actual ratings by both raters*.

**Table 3b,c:** Subjective image quality scores as given by both raters for mDixon images (3b) and subtraction images (3c) of the abdominal position.

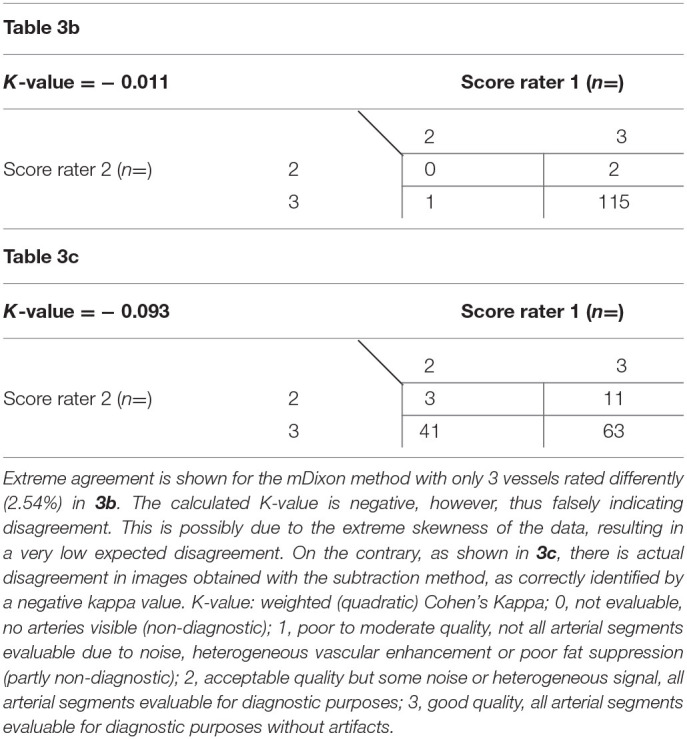

### Vessel-to-Background Analysis

**Figure 4:**
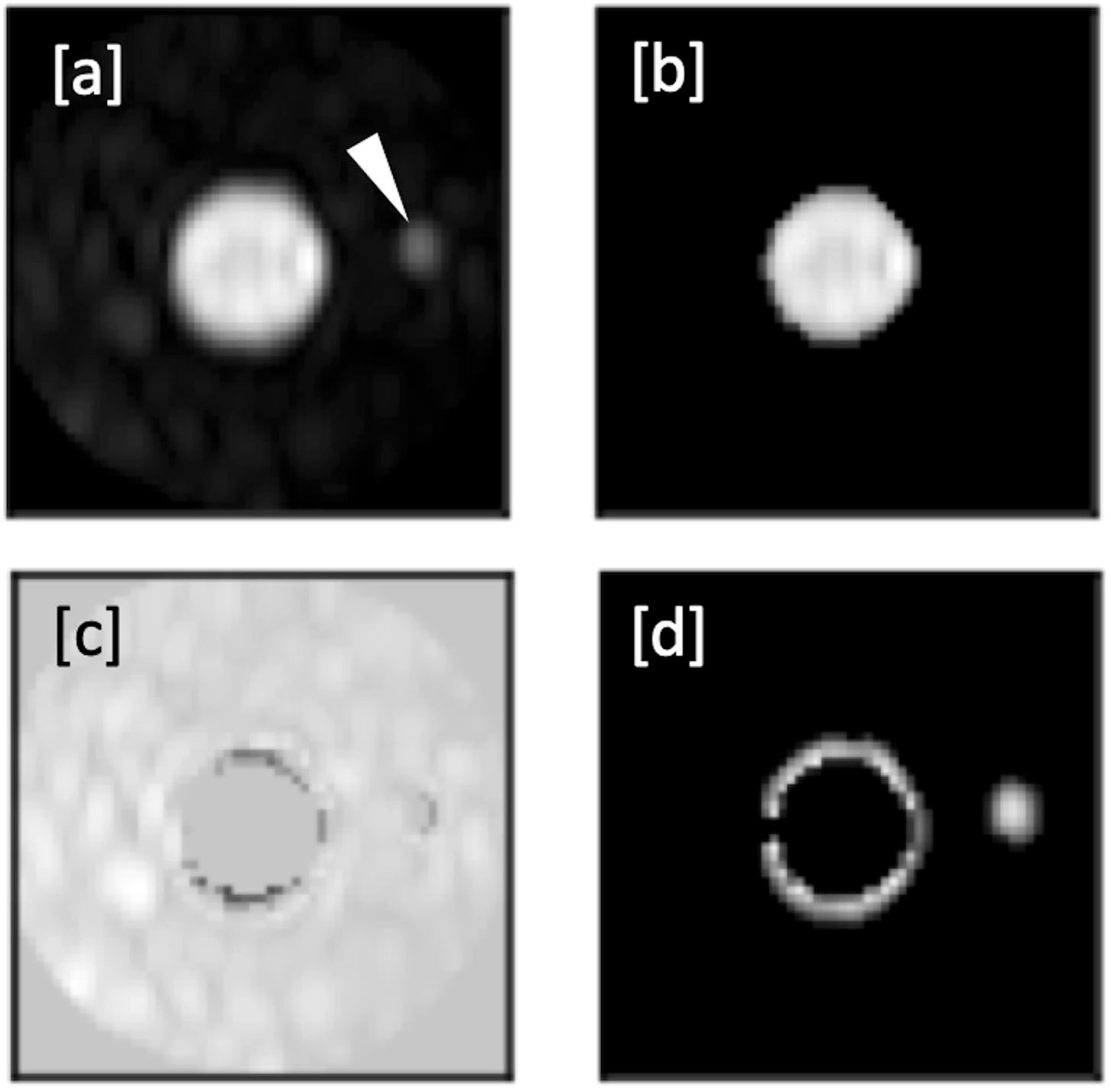
**(a–d)**: Automated segmentation of a right femoral artery in an mDixon image. **(a)** Shows the ROI with femoral artery in the center and a smaller adjacent vessel to the right (arrowhead in **a**). **(b)** Shows the full extent of the vessel as identified by the flood fill algorithm. The resulting mask used for background signal calculation is shown in **c**), whereas **d**) shows the discarded local maxima including the correctly identified secondary vessel.

ROI placement was feasible in all prespecified vessel segments. Segmentation into vessel and background was successfully carried out in all ROIs. An example of this segmentation is shown in Figures 4a–d. Overall mean VBC was significantly better in mDixon images (23.16 ± 8.4 SD in mDixon images vs. 19.00 ± 8.14 SD in subtraction images; *P* < 0.001), resulting in a gain of 22% compared with subtraction images.

The values for VBC were heterogeneous between patients and table positions, with the best results in the upper leg position, followed by the abdominal position (Table 4). The gain of vessel-to-background contrast was highest in the upper leg position, amounting to 39%. For the lower leg position both methods showed the least VBC with near identical values for both the mDixon and the conventional subtraction method (*p* = 0.91).

**Table 4:** Mean vessel to background contrast total and per anatomical location.

		**Anatomical location**		
	**Total**	**Abdominal position**	**Upper leg position**	**Lower leg position**
mDixon (mean ± SD)	23.16 ± 8.4	23.69 ± 6.8	31.33 ± 6.3	15.60 ± 4.2
Subtraction (mean ± SD)	19.00 ± 8.1	18.98 ± 8.5	22.60 ± 4.3	15.56 ± 4.4
Factor	1.22	1.25	1.39	1.00
*P*-value	<0.001	<0.001	<0.001	0.91

*SD, Standard deviation; Factor, Mean gain in vessel to background contrast with mDixon as compared to the subtraction method*.

### Signal-to-Noise Ratio Analysis

Mean gain in signal-to-noise ratio was 1.82 or 82% in the mDixon images as compared to the subtracted images. It was highest in the upper leg position (2.07) and gradually less pronounced in the lower leg position (1.95) and the abdominal position (1. 57). Detailed results are summarized in Table 5.

**Table 5:** Noise and mean SNR gain by the mDixon method as compared with the conventional subtraction method.

		**Anatomical location**		
	**Total**	**Abdominal position**	**Upper leg position**	**Lower leg position**
mDixon (mean ± SD)	102.81 ± 30.33	81.56 ± 17.3	129.69 ± 22.81	130.95 ± 17.03
Subtraction (mean ± SD)	187.11 ± 76.21	127.78 ± 27.41	253.72 ± 43.96	271.26 ± 39.98
Factor	1.82	1.57	2.07	1.95
*P*-value	<0.001	<0.001	<0.001	0.091

*SD, Standard deviation; Factor, Mean gain in signal to noise ratio with the mDixon method as compared to the subtraction method*.

## Discussion

Contrast- enhanced magnetic resonance angiography with a single dose of contrast agent using the two-point modified Dixon method is feasible at 3T and reliably provides good image quality in patients with known or suspected peripheral artery disease. We did not encounter disturbing field inhomogeneity or water-fat swapping artifacts despite the relative closeness of the TEs to in- and out-of-phase echo times at 3T (15). This potential gain of image quality with the mDixon method over the conventional subtraction method might be further appreciated when acquisition time is taken into account. Since there is no need for acquiring pre- contrast images per position anymore, examination length can be reduced significantly in comparison to subtraction imaging, which additionally renders the mDixon method less prone to motion artifacts.

**Figure 5:**
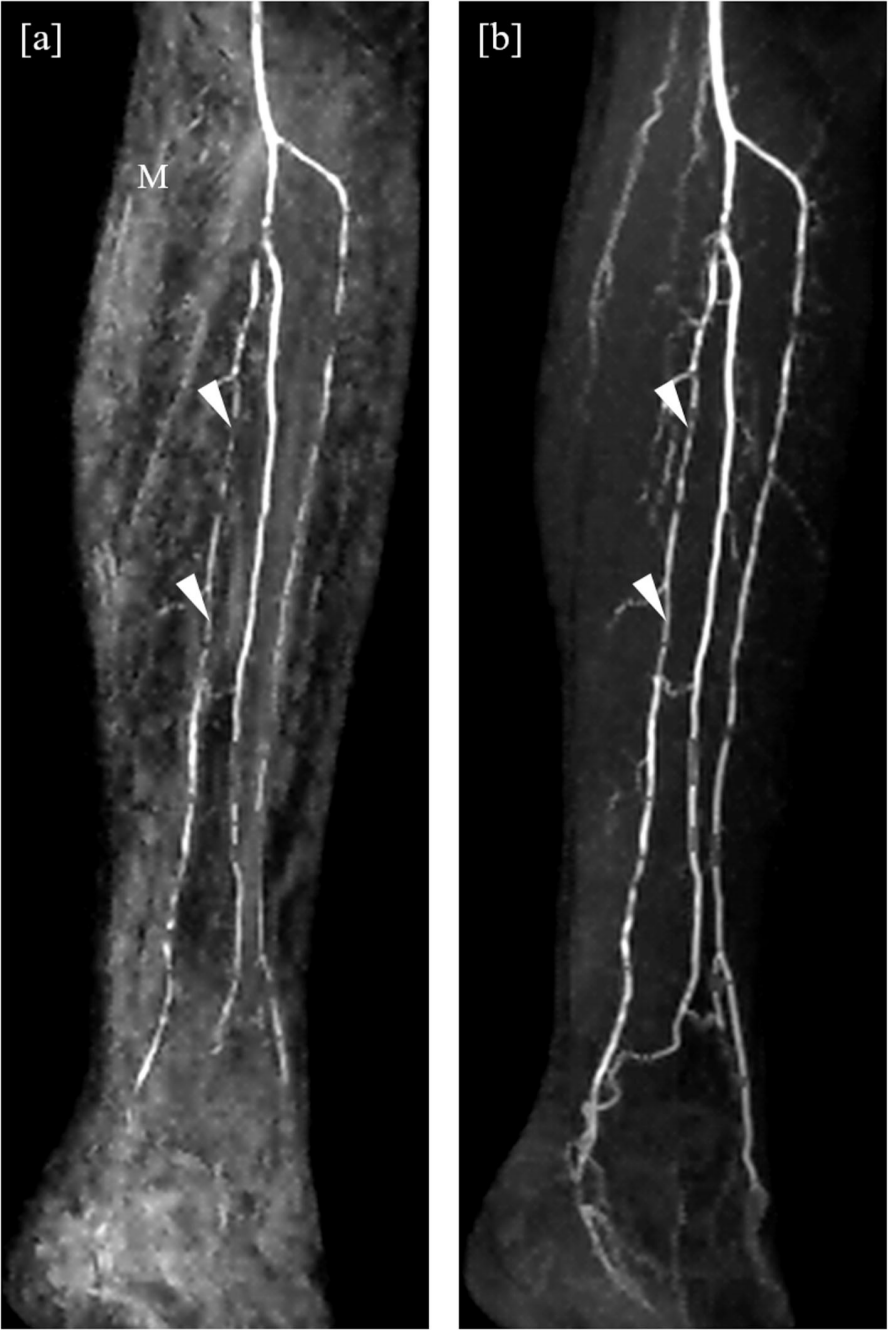
Detail of angulated coronal maximum intensity projections obtained by the subtraction method **(a)** and the subtractionless mDixon method **(b)**. Extensive misregistration artifacts (M) can be seen in the subtraction image as compared to the mDixon image, possibly due to movement of the patient. Note the better depiction of the distal arteries of the lower leg in the mDixon method. The posterior tibial artery is clearly patent, albeit stenotic, whereas it was rated inconclusive regarding occlusion in the subtraction image due to limited image quality (arrowheads).

Subjective image quality was high in our study, regardless of method, table positions, or reader. However, we found that uniformly, independent of reader or anatomical position, image quality ratings were higher with the mDixon method. A prominent advantage of the mDixon method in this regard is its elimination of misregistration artifacts. Depending on the reader, seven or ten vessel segments were considered non-diagnostic in the subtraction method in the lower leg position, but of sufficient image quality to allow stenosis assessment in the mDixon method (Figure 5). We encountered this phenomenon in subtraction images both in the upper and the lower leg position, where it was mostly due to patient movement, as well as in the abdominal position, where the motility of the internal organs reduced image quality. Nine vessels were judged to be occluded or stenotic when looking at subtraction images, but clearly patent in the mDixon images. This was likely due to artifacts in the subtraction images or to low VBC caused by noisy subtraction images. Similarly, we believe to see an overall gain in subjective image quality compared to the subtraction methods due to a better depiction of small vessels and to a residual depiction of background structures (such as anatomical landmarks) without being able to objectively measure this.

In accordance with the visually assessed image quality improvement with the mDixon method, our study showed an increase in VBC of 22% and in SNR of 82% compared to the conventional subtraction method. The measured SNR gain is in good agreement with the predicted values at the selected echo times. Semi-automated VBC evaluation was successfully carried out in all patients. We substantially reduced the expenditure and complexity of defining the true background signal surrounding a vessel by automatically eliminating adjacent vessels and other local signal intensive structures. The fact that we could not show a significant improvement for vessel-to-background contrast in the lower leg position is somewhat contradictory, given the respective improvements in subjective image quality and signal-to-noise ratio. This may in part be attributed to an increased contrast accumulation in the background tissue at the late acquisition stage in the lower leg, which generally renders the calculation of a true background signal difficult. While some, including the authors of this study, consider an elevated background signal as an advantage, because the visibility of anatomical landmarks is preserved, it may hamper the acceptance of the subtractionless approach by others. Advanced post-processing algorithms were presented (16) that permit tailoring the extent of background signal remaining in the MIPs, especially in the legs and the pelvis, to individual preferences.

In our work, the overall gain in SNR was 1.82, thus reasonably close to the theoretically possible values as described in the methods section, which supports the hypothesis that at 3T, the noise propagation in the water-fat separation in the present application is even more favorable than at 1.5T. For the desired resolution, the available gradient performance, and the predominant importance of speed, the resulting echo spacing of ~1.2–1.4 ms is much closer to the, from an SNR perspective, optimal 1/(2^*^ Δf_F_) at 3T than at 1.5T.

Our study is focussed on a direct comparison between subtraction and mDixon angiography, where acquisition times and spatial resolution were very similar to those obtained in prior studies at 1.5T (8). However, the observed gain in SNR may translate into improved spatial resolution, or increased scan acceleration factors and reduced acquisition time per station to reduce venous contamination, which merits further investigation.

The feasibility of single- dose contrast- enhanced magnetic resonance imaging for peripheral arteries has been shown before, as well as the feasibility of using the mDixon method at 1.5 T to achieve substantial improvements in image quality, VBC and SNR (8). We successfully transferred this approach to 3T with better overall image quality and interrater agreement for the mDixon method and conducted a visual and quantitative assessment of SNR, VBC and image quality in comparison to conventional subtraction angiography.

Semi-automated processes to define the extents of vessels and to derive VBC have been described before for magnetic resonance angiography (17), but to our knowledge this is the first time that such a method is successfully employed in peripheral artery magnetic resonance angiography. It allows for an analysis of large numbers of segments and subjects, improving the confidence of the results. In the present work, a total number of 690 segments have been analyzed, where at least 2 ROIs per segment are required for a conventional analysis of VBC, which would render a manual evaluation cumbersome and prone to errors.

Our study has several limitations. First of all, we did not compare the images to the gold standard digital subtraction angiography and no invasive measurement of stenosis severity was employed. Our reference method, CE-MRA, has however been proven to be a reliable imaging method with a high diagnostic accuracy for peripheral artery disease detection (3, 18, 19). To ultimately assess diagnostic accuracy of the subtractionless modified Dixon method, a direct comparison to an independent method such as invasive angiography or digital subtraction angiography would be preferable. Secondly, our protocol stipulated an identical dose of contrast agent for all patients, regardless of weight, or height. Thus, we cannot generalize our findings for weight-adjusted imaging protocols. An important limitation of our semi-automated vessel-to background analysis consists of the manual setting of ROIs, as this allows for interobserver variability. However, in direct comparison to an entirely manual approach, our method might reduce variability. Further research is necessary to fully assess its value for clinical routine. We did not measure direct SNR values and instead derived the gain in SNR with the mDixon over the subtraction method from an additional scan in a healthy volunteer. Given the multitude of approaches to measure SNR and their respective intrinsic drawbacks, we argue that by our indirect method we avoid common pitfalls in SNR calculation (10). Lastly our patient cohort comprises only 10 patients. Despite our success to show a statistically significant improvement in image quality, further studies with larger patient samples are needed.

In conclusion, this study demonstrated the feasibility of subtractionless first-pass peripheral MRA at 3T in patients with known or suspected PAD using a three- positions stepping table approach with a single dose of contrast agent and conducted a comprehensive comparison with conventional subtraction angiography. Our results indicate that the predicted increase in SNR for the given echo time selection at 3T, as well as its robustness against motion artifacts translates well into improved image quality in clinical practice.

## Data Availability Statement

The original contributions presented in the study are included in the article/supplementary material, further inquiries can be directed to the corresponding author/s.

## Ethics Statement

The studies involving human participants were reviewed and approved by ethics committee of the Charité—Universitätsmedizin Berlin. Written informed consent for participation was not required for this study in accordance with the national legislation and the institutional requirements.

## Author Contributions

HE, BS, MK, and SK conceived and planned the image acquisition. KW, CS, and MN performed the measurements. SK, PS, and BP were involved in planning and supervised the work. KW and SK processed the experimental data, performed the analysis, drafted the manuscript, and designed the figures. HE and MK implemented and optimized the modified Dixon MRA technique. CS implemented the algorithms for semiautomated calculations. All authors discussed the results and commented on the manuscript.

## Conflict of Interest

HE was employed by the company Philips Research, Hamburg, Germany. CS and BS were employed by the company Philips Healthcare, Hamburg, Germany. MK was employed by the company Philips Healthcare, Best, Netherlands. The remaining authors declare that the research was conducted in the absence of any commercial or financial relationships that could be construed as a potential conflict of interest.

## Abbreviations

CE-MRA: Contrast-enhanced magnetic resonance angiography
PAD: Peripheral artery disease
mDixon: modified Dixon
MR: Magnetic resonance: MRA, Magnetic resonance angiography
SNR: Signal-to-noise ratio
VBC: Vessel-to-background contrast
T: Tesla
TE: Echo time
TR: Repetition time
ROI: Region of interest
Δf_F_: Offset of resonance frequency of fat relative to water
FOV: Field of view
RF: Radiofrequency
SENSE: Sensitivity encoding
MIP: Maximum intensity projection.
